# Complementary Strategy Enhancing Broad-Specificity for Multiplexed Immunoassay of Adulterant Sulfonylureas in Functional Food

**DOI:** 10.3390/bios12080591

**Published:** 2022-08-02

**Authors:** Zhaodong Li, Haihuan Xie, Tingdan Fu, Yingying Li, Xing Shen, Xiangmei Li, Yi Lei, Xiaojun Yao, Anastasios Koidis, Yingju Liu, Xinan Huang, Hongtao Lei

**Affiliations:** 1 Guangdong Provincial Key Laboratory of Food Quality and Safety/National-Local Joint Engineering Research Center for Machining and Safety of Livestock and Poultry Products, South China Agricultural University, Guangzhou 510642, China; scaulizhaodong@scau.edu.cn (Z.L.); shenxing325@163.com (X.S.); lixiangmei12@163.com (X.L.); yingjuliu@scau.edu.cn (Y.L.); 2 Wuzhou Institute for Food and Drug Control, Wuzhou 543099, China; 704596242@foxmail.com; 3 Institute of Tropical Medicine, Guangzhou University of Chinese Medicine, Guangzhou 510400, China; tingsane@163.com; 4 Wuzhou Product Quality Inspection Institue, Wuzhou 543001, China; liyingyingwz@163.com; 5 Guangdong Institute of Food Inspection, Zengcha Road, Guangzhou 510435, China; leiy04@foxmail.com; 6 Dr. Neher’s Biophysics Laboratory for Innovative Drug Discovery, State Key Laboratory of Quality Research in Chinese Medicine, Macau Institute for Applied Research in Medicine and Health, Macau University of Science and Technology, Macau 999078, China; xjyao@must.edu.mo; 7 Institute for Global Food Security, Queen’s University Belfast, 19 Chlorine Gardens, Belfast BT9 5DJ, UK; t.koidis@qub.ac.uk; 8 Tropical Medicine Institute & South China Chinese Medicine Collaborative Innovation Center, Guangzhou University of Chinese Medicine, Guangzhou 510405, China; xinanhuang@gzucm.edu.cn

**Keywords:** sulfonylureas, broad-specificity, antibody, immunoassay, adulteration, functional foods

## Abstract

Sulfonylureas, a family of anti-diabetic drugs widely used in the clinical treatment of type 2 diabetes, have recently emerged as an illegal adulterant in functional foods, to enhance the claimed anti-diabetic activity. To establish a screening assay method against their adulteration, with the aid of molecular simulation of hapten, two antibodies were raised and complementarily used to enhance the broad-specificity of an enzyme-linked immunosorbent assay (ELISA), which demonstrated simultaneous detection capability to 6 sulfonylureas; the detection limits ranged from 0.02 to 1.0 ng/mL, and recoveries were between 78.3% to 104.5%. Liquid chromatography with tandem mass spectrometry (LC-MS/MS) confirmed the reliability of the proposed ELISA, based on real samples. These results suggest that the proposed ELISA could be an ideal method for screening to monitor for illicit adulteration of sulfonylureas in functional pill products.

## 1. Introduction

More than 463 million people worldwide are afflicted by a chronic disease characterized the persistent hyperglycemia known as type 2 diabetes [1]. These patients need to stay on medication throughout their lives to help their bodies metabolize glucose. The common hypoglycemic drugs include insulin, metformin, and sulfonylureas. However, the body may develop resistance if these medications are given in the long term [2]. As a result, to reduce dependence on glucose-lowering drugs, many diabetics prefer to consume functional foods or natural medicines that are supposedly “natural, side-effect-free, and efficacious” [3]. However, some so-called strongly active products have recently been found to be illegally added synthetic drugs, such as sulfonylureas, added to functional foods or natural medicines [4] in order to “boost” their effect.

Sulfonylureas are a typical class of drugs used orally for the treatment of type 2 diabetes, including first-generation drugs that are no longer used (acetohexamide, tolbutamide, chlorpropamide, carbutamide, and tolazamide), and second-generation drugs (gliclazide, glyburide, glipizide, gliquidone, glimepiride, and glibornuride) [5]. The addition of these therapeutic drugs to dietary supplements illegally violates the law and the original intent of consumers. However, on a more important note, it will lead to a profound and serious public health problem, exposing consumers to the risk of serious adverse reactions in the form of acute hypoglycemia, diarrhea, and erythrocytic anemia [6]. Currently, analytical methods for sulfonylureas are generally instrumental methods, such as ultra-performance liquid chromatography (UPLC) [7], liquid chromatography-tandem mass spectrometry (LC-MS/MS) [8], real-time mass spectrometry (DART-MS) [6,9], and on-line two-dimensional liquid chromatography (2D-MS) [10], etc. Instrumental methods are very sensitive and reliable. However, they require complex sample pretreatment, expensive equipment support, and large amounts of organic solvents that cannot be used for the rapid detection of sulfonylureas in functional foods. In contrast, immunoassay, such as the enzyme-linked immunosorbent assay (ELISA), has been widely used in the rapid screening of illegal additives in functional foods, due to its simplicity, rapidity, and ease of interpretation [11,12]. However, no ELISA has been reported for sulfonylurea detection in functional foods. Besides, it is known that the sulfonylurea family contains a class compound with similar structures and activity; therefore, there is an urgent need to enhance assay efficiency to establish a immunoassay that possesses the simultaneous detection capability for sulfonylureas in one, rather than repeatedly.

**Figure 1:**
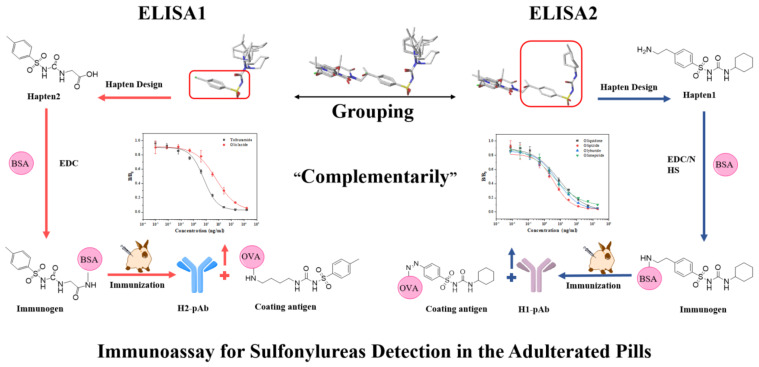
Schematic illustration of complementary ic-ELISA for Sulfonylureas. H1-pAb, antibody to Hapten 1. H2-pAb, antibody to Hapten 2. BSA, bovine serum albumin. EDC/NHS, two coupling regents EDC and NHS for active ester method.

In this study, four novel haptens were rationally designed by aligning the common structure of molecular modeling of 11 sulfonylureas, and then immunizing rabbits to generate two polyclonal antibodies with broad-specificity that can recognize many sulfonylureas. An ELISA (Figure 1) was established based on a complementary strategy, the combination of the obtained two antibodies for the simultaneous detection of 6 sulfonylureas, which has recently been in functional pills with claimed anti-diabetic properties.

## 2. Materials and Methods

### 2.1. Chemicals and Reagents

The chemicals 4-(2-aminoethyl) benzenesulfonamide (CAS 35303-76-5), ethyl tosylcarbamate (CAS 5577-13-9), di-tert-butyl decarbonate (CAS 24424-99-5), 4-methylbenzenesulfonyl isocyanate (CAS 4083-64-1), cyclohexyl isocyanate (CAS 3173-53-3), tert-butyl (4-aminobutyl)carbamate (CAS 1088779-66-1), glycine methyl ester hydrochloride (CAS 5680-79-5), N-(4-sulfamoylphenyl)acetamide (CAS 121-61-9), acetohexamide, tolbutamide, chlorpropamide, carbutamide, tolazamide, glibornuride, gliclazide, glimepiride, glyburide, gliquidone, glipizide, rosiglitazone, phenformin, metformin hydrochloride, and repaglinide, were purchased from Shanghai Aladdin Biochemical Technology Co., Ltd. (Shanghai, China). Bovine serum albumin (BSA), ovalbumin (OVA), 1-(3-Dimethylaminopropyl)-3-ethyl carbodiimide hydrochloride (EDC), hydrolyzed protein, N-hydroxylsuccinamide (NHS), Freund’s complete adjuvant, Freund’s incomplete adjuvant, 3,3′,5,5′-tetramethylbenzidine (TMB), and goat anti-rabbit IgG-HRP were purchased from Sigma-Aldrich (St. Louis, MO, USA). Dichloromethane, trifluoroacetic acid, N, N-Dimethylformamide (DMF), Tween-20 and methanol, were bought from Damao Chemical Reagent Co., Ltd. (Tianjin, China). All reagents are in analytical reagent grade or higher purity.

New Zealand White rabbits (2–3 months old about 2 kg) were purchased from Guangdong Medical Laboratory Animal Center and kept in the Animal Experiment Centre of South China Agriculture University (Animal Experiment Ethical Approval Number: 2020e009, Supplementary Materials Figure S9). All necessary animal work licenses were obtained prior to the start of the work.

### 2.2. Instruments

Ultraviolet−visible spectra were documented on a Nanodrop 2000C spectrophotometer (Thermo, MA, USA). Absorbance was measured on a Multiskan Spectrum (Thermo, Waltham, MA, USA). Polystyrene 96-well plates (KE-96−8) were acquired from Yijiamei (Xiamen, China). The LC-MS/MS experiment was performed on a AB QTRAP4500 triple quadrupole mass spectrometer (AB SCIEX, Framingham, MA, USA), and POROSHELL HPH-C18 (2.1 × 150 mm, 4 µm, Agilent, Santa Clara, CA, USA) was used to separate compounds.

### 2.3. Buffers and Solutions

The following buffers and solutions were used: (A) 0.1 mol/L 2-(N-morpholine) ethanesulfonic acid solution, pH 4.5–5.0, as binding buffer for the EDC method. (B) 0.1 mol/L carbonate buffer (CB), pH 9.6, as coating buffer. (C) Washing solution consisting of PBST solution in phosphate-buffered saline (PBS, 0.01 mol/L, pH 7.4) containing 0.5% Tween-20. (D) Blocking buffer to be prepared with 0.1% hydrolyzed protein (*w*/*v*) and 0.01 mol/L PBST. (E) 2 mol/L sulfuric acid as the termination solution. (F) Standard stock solution (1 mg/mL) produced by dissolving an appropriate amount of standards in methanol and stored at 4 °C until use.

### 2.4. Conjugation

The hapten synthesis is described in detail in the Supplementary Materials.

#### 2.4.1. Hapten 1-OVA/BSA

Hapten 1 was coupled to BSA and OVA by a direct EDC method [13] with a slight change. Briefly, 8 mg Hapten 1, 6 mg EDC, and 12 mg BSA or OVA were solubilized in 1000 µL of conjugation buffer (2-(N-morpholine) ethanesulfonic acid solution, 0.1 mol/L, pH 4.5–5.0). The solution was stirred at 4 °C for 12 h. Finally, the prepared conjugates were purified by dialysis with PBS (0.01 M, pH 7.4) at 4 °C for 3 days to remove unreacted reactants and non-coupled free hapten.

#### 2.4.2. Hapten 2-OVA/BSA

The Hapten 2-OVA/BSA was prepared using the active ester method [13]. Briefly, 3 mg Hapten 2, 5.7 mg EDC, and 1.7 mg NHS were solubilized in 200 μL of DMF. After reacting at 4 °C overnight, the activated hapten solution was added dropwise to 2 mL solution of 12 mg BSA or OVA in PBS (0.01 M, pH 7.4) under stirring and reacted for another 4 h at room temperature. The prepared conjugates were purified by dialyzing with 0.01 M PBS (pH 7.4) for 3 days at 4 °C.

#### 2.4.3. Hapten 3-OVA

The coupling method is the same as that of hapten 1-OVA/BSA, with hapten 1 changed to an equal mole of hapten 3.

#### 2.4.4. Hapten 4-OVA/BSA

The preparation of SUs4-OVA/BSA was carried out by the diazotization method. All procedures described below were performed at 0~4 °C. Six miligrams of hapten 4 were dissolved in 1 mL of methanol and adjusted to a pH of 1 with pre-cooled 1 mol/L HCl. To the solution of hapten 4 was added dropwise 0.5 M sodium nitrite, with constant stirring until the starch potassium iodide test paper turned blue and black, then continuing to react for 40 min in the dark, and obtaining a dark yellow turbid liquid. To remove the excess nitrous acid, the pH of the solution was adjusted to 8 with 3 M NaOH. The diazonium salt solution of hapten 4 was added dropwise to 12 mg of OVA or BSA in 3 mL of PBS (0.01 M, pH 7.4), and the pH of the solution was maintained at around 9.5 with 3 M NaOH. The solution was stirred for 12 h after the addition of the final drop. The prepared conjugates were purified by dialyzing with 0.01 M PBS (pH 7.4) for 3 days at 4 °C.

A full wavelength UV-Vis spectroscopy scan was applied to verify the conjugation (Supplementary Materials Figure S4) and final preservation at −20 °C until use. Hapten 1-BSA and Hapten 2-BSA Hapten 4-BSA were used as the immunogen, while Hapten 1-OVA, Hapten 2-OVA, Hapten 3-OVA, and Hapnten 4-OVA were used as the coating antigens.

### 2.5. Antibody Generation

The immunization process in animals was described in our former publication [14]. The collected antisera were assessed by ELISA, with titer and inhibition rate [15] and antisera purified by ammonium sulfate precipitation for use in further process development [16]. The purified antibodies were characterised by sodium dodecyl sulfate-polyacrylamide gel electrophoresis (SDS-PAGE) and named as H1-Ab (immunized by Hapten 1-BSA) H2-Ab (immunized by Hapten 2-BSA), and H4-Ab (immunized by Hapten 4-BSA).

### 2.6. ELISA Procedure

The detection method for sulfonylureas was made up of two independent ELISAs established by H1-Ab and H2-Ab and the corresponding coating antigen. The individual ELISA methods were the same as the general ELISA method, which is described in detail in the Supporting Information.

An inhibition rate (B/B_0_ × 100) was utilized to evaluate the binding ability of the antibody to the coating agent and sulfonylureas, where B0 and B represent the absorbance values of the negative solution and sulfonylureas standard solution (1 µg/mL), respectively. Then, a four-parameter logistic function plotted by Origin 8.5 (Origin Lab Corp., Northampton, MA, USA) was fitted to establish calibration curves of 11 sulfonylureas under optimized antibody/coating antigen concentration. The detection limit (LOD) was defined as the inhibitory concentration at 10% (IC_10_), while the dynamic detectable range was defined as the values ranging from IC_20_–IC_80_.

### 2.7. Specificity

The specificity of the ELISA was determined by the half inhibitory concentration (*IC*_50_) of the rosiglitazone, phenformin, metformin hydrochloride, repaglinide, acetohexamide, tolbutamide, chlorpropamide, carbutamide, tolazamide, gliclazide, glyburide, glipizide, gliquidone, glimepiride, and glibornuride under optimized conditions. The cross-reactivity (*CR*) was calculated according to the following equation:

(1) $$CR\left(\%\right)=\frac{IC_{50}\left(most\;sensitivity\;SUs,\;\mathrm{nmol}/\mathrm{mL}\right)}{IC_{50}\left(other\;SUs,\;\mathrm{nmol}/\mathrm{mL}\right)}\times 100\%$$

### 2.8. Sample Preparation

Healthcare capsules were acquired from the locally available market and determined to be “true negative” by LC-MS/MS (see Section 2.10, Recovery). The shell of the capsule samples was removed to collect the powder. For each sample, 1.000 g of the sample powder was accurately weighed into a 15 mL centrifuge plastic tube, 4 mL of methanol was added and vortex-mixed for 2 min, centrifuged at 4000× *g* for 5 min, and the supernatant was aspirated to obtain the sample’s working solution. For the tablet sample, the powder was obtained by grinding in a mortar, and the remaining treatment was the same as the capsule.

### 2.9. Matrix Effect

Blank capsule samples were prepared as described above. The working solutions of the samples were diluted to 40, 60 and 80 times with PBST, respectively. Glipizide (for H1-Ab ELISA) or tolbutamide (for H2-Ab ELISA) was then diluted with PBST and three different dilution extracts to establish calibration curves for the ELISA. To ensure that matrix effects were negligible, *t*-tests were used to compare calibration curves and confirm the most appropriate dilution.

### 2.10. Recovery and Confirmation

Recovery is the addition of a standard of known content, i.e., the component being measured, to a blank sample or to a background of known content, and the ratio of the measured value to the added value calculated using an established method. Six sulfonylureas, often employed as illicit additions, were spiked in capsule samples and compared with the LC-MS/MS method to evaluate the detection accuracy of the developed ELISA. The capsule samples were added to three concentration levels of glyburide (0.32, 1.6, and 6.4 mg/kg), glipizide (0.32, 1.6, and 4.8 mg/kg), glimepiride (0.32, 1.6, and 6.4 mg/kg), gliquidone (0.64, 3.2, and 12.8 mg/kg), tolbutamide (0.32, 1.6, and 6.4 mg/kg), and gliclazide (3.2, 16, and 64 mg/kg), respectively. Three replicates were tested. The samples were then subjected to the extraction method described above. The accuracy and precision were estimated using the recovery and coefficient of variation (CV), respectively. The determination coefficient (R^2^) between ELISA and LC-MS/MS method was used to evaluate the reliability of the ELISA.

The standard tolbutamide, glyburide, glipizide, glimepiride, gliquidone, and gliclazide were dissolved in MeOH with a concentration from 1.0~1000.0 ng/mL, respectively. As mentioned in a previous article [17], LC-MS/MS analysis was performed on AB QTRAP4500 triple quadrupole mass spectrometer.

(2) $$\mathrm{recovery}\left(\%\right)=\frac{Detected\;concentration}{Actual\;concentration\;added}\times 100\%$$

### 2.11. Analysis of Blind Samples

**Figure 2:**
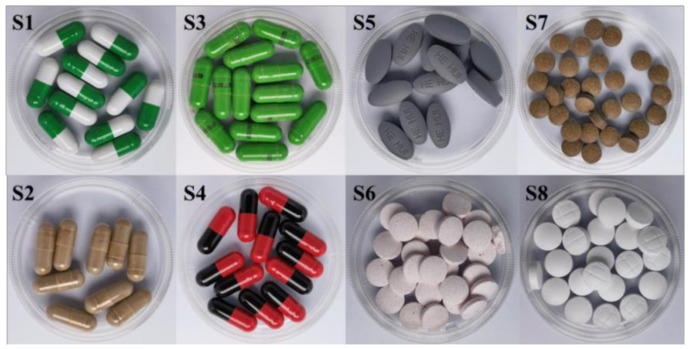
Eight real samples claiming anti-diabetic properties. S1~S4 were capsule samples with different matrices. S5~S8 were tablet samples with different matrices.

Eight samples (Figure 2) were purchased from the local market, after being prepared by the above extraction method, and the extract was diluted at appropriate concentration; the extraction was then analyzed by both the established ELISA and LC-MS/MS simultaneously.

## 3. Results

### 3.1. Antibody Production

#### 3.1.1. Hapten Design

**Figure 3:**
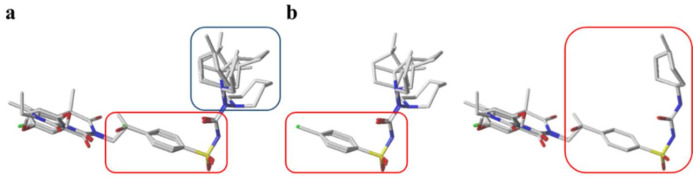
The alignment result of 11 sulfonylureas with the lowest energy conformations. (**a**) total alignment result of gliquidone, glipizide, acetohexamide, glyburide, glimepiride, tolazamide, tolbutamide, chlorpropamide, gliclazide, carbutamide, and glibornuride. (**b**) grouping alignment result. The left side included tolbutamide, carbutamide, gliclazide, tolazamide, glibornuride, and chlorpropamide; The right side included glyburide, glipizide, glimepiride, gliquidone, and acetohexamide. Blue and red rectangular boxes represent the variant and the common skeleton structure of sulfonylureas.

Hapten design plays a key role in the whole process of producing a broad-specificity antibody and developing a broad-specificity immunoassay. To obtain the broad-specific antibody, that can widely recognize a group of serial compounds with different but usually related structures [18], structural similarity is often considered the basis of immune hapten selection that induces broad-specific antibody production. For the structural analysis of 11 sulfonylureas, it was found that the molecular structures can be divided into three parts, R1, R2, and general structural (S-arylsulfonylurea) (Table 1). Further analysis showed that the 11 sulfonylureas can be further grouped into two parts. One part is R2 with cyclohexane (named Group 1), while the other is R1 with the single-atom substituent (named Group 2). In addition, from the alignment result of 11 sulfonylureas (Figure 3), grouping can significantly reduce group structure differences, indicating that designing a broad-specificity hapten for each group will help improve the total affinity of broad-specificity antibodies. Thus, Hapten 1–4 was designed (Table 1). Hapten 1 and Hapten 3 retain the S-arylsulfonylurea and cyclohexane features of Group 1. Besides, the p-substituent on the phenyl ring of Hapten 1 was ethylamine, which could be coupled to BSA by forming an amide bond with the one-step EDC method. The amide bond-forming was consistent with most of the structures in Group 1. To improve the affinity of the antibody prepared with the Hapten 1 to Group 1. Hapten 3 is designed as a heterogeneous coating hapten to reduce the influence of “linking arm antibodies” [19], thereby increasing the detection sensitivity to the ELISA process. The design of Hapten 2 and Hapten 4 was similar to that of Hapten 1 and Hapten 3. However, since it is hard to determine which structure is more suitable for antibody preparation, both Hapten 2 and Hapten 4 were used as immunogens synthesis and as heterogeneous coating haptens to each other.

#### 3.1.2. Conjugate Preparation

A UV−vis spectrophotometer was utilized to determine the effectiveness of the conjugation reaction of Hapten 1-OVA/BSA, Hapten 2-OVA/BSA, Hapten 3-OVA, and Hapten 4-OVA/BSA. As shown in Figure S10a,b,e,f, the proteins modified with Hapten 1 and Hapten 4 demonstrated a slight shift in the valley at 250 nm, compared with the natural protein. Furthermore, Hapten 1 and 4 also demonstrated a pronounced absorption peak at 240 nm. Therefore, the shift of the UV absorption peak of the protein can be ascribed to the successful coupling of the hapten and carrier protein [20]. Similarly, Hapten 2-OVA/BSA (Figure S10c,d), due to the active ester method, is more effective than the direct EDC method; the conjugation showed a significant shift compared with the natural protein. While the Hapten 4 coupled to the BSA/OVA by the diazotization method (Figure S10g,h), the formation of diazonium bonds caused Hapten 3-BSA/OVA to display a new absorption peak at 350 nm. This indicated that Hapten 4 was successfully coupled to the carrier protein [20].

#### 3.1.3. ELISA Optimization

To obtain a high sensitivity, 12 combinations with three antibodies and four coating antigens were evaluated by titer and inhibition rate. The result (Figure S11) shows that the combinations of H1-Ab/Hapten 3-OVA and H2-Ab/Hapten 4-OVA had a superior inhibition rate and higher titer, which were 83.3%/128,000 and 88.8%/256,000, respectively. Therefore, the heterologous assay combinations of H1-Ab/Hapten 3-OVA and H2-Ab/Hapten 4-OVA were selected for further optimization.

It is well known that the concentration of the coated antigen and antibody could affect the sensitivity of an immunoassay [13]. Thus, a chessboard titration was used to optimize the dilute multiple of coating antigen and purified antibody (Figure S13), by comparing the values of the IC_50_ and Amax/IC_50_ ratio (Figure S13), calculated from the calibration curve [14], where Amax represents the maximum signal at zero calibrator concentration. The optimal dilution multiple of coating antigen and antibody for H1-Ab/Hapten 3-OVA and H2-Ab/Hapten 4-OVA were found to be 1:32,000/1:512,000 and 1:64,000/1:4000, respectively.

#### 3.1.4. Broad-Specificity

The specificity of the two antibodies was evaluated by cross-reactivity of 11 sulfonylureas and four other oral hypoglycemic drugs usually adulterated in the capsule (Table 2); the H1-Ab and H2-Ab showed a desirable broad-specificity with sulfonylureas and no obvious cross-reactivity (CR) for other drugs. Since hapten 1 and 3 bind to the protein at R1 and hapten 2 and 4 bind to the protein at R2, the R1 terminus of glimepiride, glipizide, glyburide, gliquidone, and acetohexamide are similar and complex, while the R2 terminus is essentially the same. Therefore, the attachment of the protein at the R1 end exposes the common R2 end, so that antibodies immunized with hapten 1 recognize all five drugs simultaneously. Their IC_50_ were 7.1, 8.5, 12.1, 14.8 and 29.4 nmol/µL, and their LOD (limit of detection) were 0.1, 0.7, 0.4, 0.04, and 2.1 nmol/µL, respectively. Conversely, tolbutamide, chlorpropamide, and gliclazide have similar and complex R2 ends and essentially identical R1 ends, so that the attachment of the protein at the R2 end exposes the common R1 end and therefore the antibody from semi-antigen 2 recognizes all three drugs simultaneously. The IC_50_ of tolbutamide, chlorpropamide, and gliclazide were 23.3, 33.6 and 179.6 nmol/µL, respectively, and 4, 23, and 4 times more sensitivity than that of H1-Ab. Hence, these data indicated the effectiveness of the hapten design and proved that this “complementary” format (combination of H1-Ab and H2-Ab) could be used to establish a more broad-specific ELISA.

### 3.2. Matrix Effect

**Figure 4:**
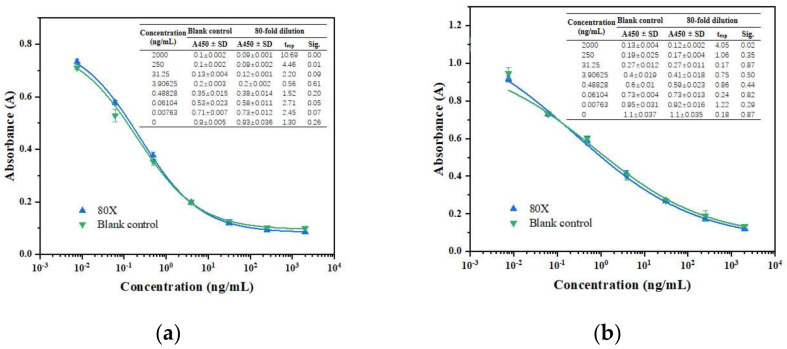
Calibration curves and *t*-test results of ic-ELISA in PBST and dilution 80 times (**a**) Glipizide as the drug for H1-Ab/Hapten 3-OVA ic-ELISA; (**b**) Tolbutamide as the drug for H2-Ab/Hapten 4-OVA ic-ELISA; The texp and Sig. determined by the *t* test (*p* = 0.05).

The two ELISA methods were evaluated for their matrix effect by comparing the calibration curve of gliquidone or tolbutamide in PBST, which was used as a control with those calibration curves of gliquidone or tolbutamide in capsule extracts (Figure 4). The *t*-test results showed that the significance values of 80 times dilution were more than 0.05, within the dynamic working range, indicating that no significant differences were encountered between the two matrixes at the 0.05 level. This indicated that the matrix effect could be negligible with 80 times dilution of the capsule extracts. Considering sample preparation and the matrix effect, the LODs for glimepiride, glipizide, glyburide, gliquidone, tolbutamide, and gliclazide were respectively 16.0, 86.4, 70.4, 4.48, 137.6, and 316.8 μg/kg, lower than the LODs of the statutory instrument detection method established by China [21]. Hence, the proposed ELISA can be used to detect both spiked and authentic samples.

### 3.3. Recovery and Confirmation

The ELISA recoveries for the six sulfonylureas ranged from 79.2% to 110.0%, with the CVs ranged from 3.7% to 18.5% (Table 3), indicating that the developed ELISA method for six sulfonylureas possessed an acceptable accuracy and excellent reproducibility. The recoveries of LC-MS/MS ranged from 76.4% to 112.1%, with CVs of 0.4% to 10.4%. In addition, the R2 ranged from 0.978 to 0.999, which indicated a good consistency between ELISA and LC-MS/MS.

### 3.4. Analysis of Blind Samples

Analysis of blind samples can verify the adaptability of the established method in different pill matrices. As displayed in Table S2, all samples were sulfonylureas free, and this may be due to fewer or no cases of sulfonylurea adulteration in functional foods recently, or a limitation of the small number of samples collected. However, this does not mean that sulphonylureas are not added to functional foods. Sulphonylureas are still a potential risk for illegal addition to functional foods [22]. Sampling of functional foods for sulphonylureas will continue in follow-up work. It was also found that there were no false positive results for the detected eight samples, which indicates that the developed ELISA methods may be reliably used for different kinds of pills claiming anti-diabetic properties.

## 4. Conclusions

To summarize, a high sensitivity ELISA was developed using two anti-sulfonylureas antibodies with broad-specificity, based on a complementary strategy. The developed method could be used for the screening assay of illegally added sulfonylurea drugs in pills with a simple methanol extraction and dilution, demonstrating consistency with results obtained using the LC-MS/MS method. The proposed method could also potentially be used for multi-analysis of sulfonylureas in other similar products.

## Figures and Tables

**Table 1:** Structures and classification of 11 sulfonylureas and the structures of 4 designed hapten.

Name	General Structural	R_1_^a^	R_2_^b^	Sulfonylureas	R_1_	R_2_
Glyburide	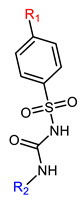	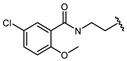	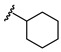	Tolbutamide	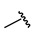	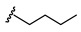
Glipizide		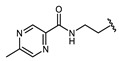	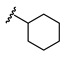	Carbutamide	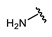	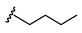
Glimepiride		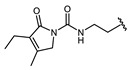	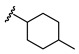	Gliclazide	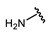	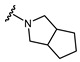
Gliquidone		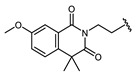	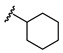	Tolazamide	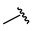	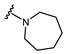
Acetohexamide		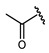	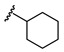	Glibornuride	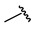	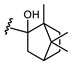
				Chlorpropamide	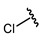	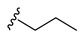
Hapten	Hapten 1	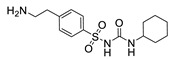		Hapten 2	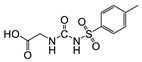	
	Hapten 3	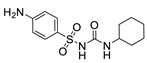		Hapten 4	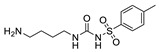	

^a^ R1, The upper part of a structure common to sulfonylureas and connected by wavy lines; ^b^ R2, The lower part of the structure common to sulfonylureas and connected by wavy lines.

**Table 2:** Specificity and sensitivity of the ic-ELISA.

Name	H1-Ab				H2-Ab			
	LOD ^a^ nmol/μL	Dynamic Working Range (nmol/μL)	IC_50_ ^b^ nmol/μL	CR ^c^ %	LOD nmol/μL	Dynamic Working Range nmol/μL	IC_50_ nmol/μL	CR %
Glimepiride	0.1	0.6–102.5	7.1	100.0	1672.4	-	17,228.5	0.1 ^d^ (0.04) ^e^
Glipizide	0.7	1.6–45.8	8.5	83.6	1823.3	-	>10,000	<0.01
Glyburide	0.4	1.6–96.4	12.1	58.7	3788.8	-	>10,000	<0.01
Gliquidone	0.04	1.1–190.7	14.8	48.3	2455.7	-	>10,000	<0.01
Acetohexamide	2.1	5.3–161.9	29.4	24.2	23.5	-	4173.0	0.6 (0.2)
Tolazamide	7.7	19.9–530.5	103.1	6.9	2.2	11.2–3221.6	189.5	12.3 (3.8)
Tolbutamide	32.2	81.7–1947.8	398.7	1.8	1.5	4.4–127.2	23.3	100 (30.6)
Carbutamide	66.3	161.1–3331.6	732.3	1.0	15.8	62.7–6911.3	658.6	3.5 (1.1)
Gliclazide	37.7	115.3–5224.3	775.8	0.9	3.1	13.9–2347.8	179.6	13.0 (4.0)
Chlorpropamide	80.2	267.8–16,444.3	2098.7	0.3	0.7	6.9–163.7	33.6	69.3 (21.2)
Glibornuride	228.1	-	>10,000	<0.01	9.8	52.4–15,498.0	901.0	2.6 (0.8)
Repaglinide	-	-	>10,000	<0.01	-	-	>10,000	<0.01
Rosiglitazone	-	-	>10,000	<0.01	-	-	>10,000	<0.01
Phenformin	-	-	>10,000	<0.01	-	-	>10,000	<0.01
Metformin hydrochloride	-	-	>10,000	<0.01	-	-	>10,000	<0.01

^a^ LOD, detection limit. ^b^ IC_50_, half inhibitory concentration. ^c^ CR, cross-reactivity of H1-Ab was calculated according to IC_50_ (other SUs) divided by IC_50_ (glimepiride). ^d^ CR of H1-Ab calculated with the IC_50_ (tolbutamide). ^e^ CR of H1-Ab calculated with the IC_50_ (glimepiride).

**Table 3:** Recovery of sulfonylureas in capsule by ELISA and LC-MS/MS (*n* = 3).

Name	Spiked Level (μg/kg)	ELISA			LC-MS/MS			R^2 b^
		Observed Level (μg/kg)	Average Recovery %	CV ^a^ %	Observed Level (μg/kg)	Average Recovery %	CV %	
Glipizide	320	259.5	81.1	10.9	244.3	76.4	1.0	0.995
	1600	1272.0	79.5	7.9	1543.5	96.5	0.8	
	4800	4434.7	92.4	4.1	4701.4	98.0	2.4	
Glimepiride	320	318.8	99.6	10.8	253.5	79.2	9.4	0.994
	1600	1548.8	96.8	18.5	1340.3	83.8	3.1	
	6400	5526.3	86.3	14.2	5745.4	89.8	1.6	
Gliquidone	640	703.8	110.0	12.4	647.8	101.2	0.4	0.995
	3200	3092.0	96.6	5.7	3366.3	105.2	2.1	
	12,800	11,472.8	89.6	7.4	12,304.1	96.1	1.8	
Glyburide	320	301.9	94.3	6.8	267.4	83.6	10.4	0.978
	1600	1554.4	97.1	6.2	1409.5	88.1	1.3	
	6400	5621.5	87.8	3.7	6363.2	99.4	2.8	
Tolbutamide	320	319.2	99.7	10.4	315.9	98.7	5.8	0.999
	1600	1550.3	96.9	9.0	1722.5	107.7	0.9	
	6400	6494.9	101.5	6.5	6539.4	102.2	0.7	
Gliclazide	3200	2534.1	79.2	18.1	3013.1	94.2	2.7	0.994
	16,000	16,034.9	100.2	6.8	14,888.0	93.1	1.1	
	64,000	67,399.1	105.3	4.1	71,709.9	112.1	3.8	

^a^ CV, coefficient of variation. ^b^ R^2^, determination coefficient.

## Data Availability

Not applicable.

## Supplementary Materials

The following supporting information can be downloaded at: https://www.mdpi.com/article/10.3390/bios12080591/s1, Figure S1: The synthetic routes of Hapten 1; Figure S2: The result of ESI-MS analysis (negative) for Hapten1; Figure S3: The synthetic routes of Hapten 2; Figure S4: The result of ESI-MS analysis (negative) for Hapten 2; Figure S5: The synthetic routes of Hapten 3; Figure S6: The result of ESI-MS analysis (negative) for Hapten 3; Figure S7: The synthetic routes of Hapten 4; Figure S8: The result of ESI-MS analysis (negative) for Hapten 4; Figure S9: Ethical review of animal experiments; Figure S10: The UV-VIS spectroscopy of Hapten1-BSA (a), Hapten1-OVA (b), Hapten2-BSA (c), Hapten2-OVA (d), Hapten3-BSA (e), Hapten3-OVA (f), Hapten4-BSA (g), Hapten4-OVA (h), and corresponding carrier protein and hapten; Figure S11: Titer and inhibition test for H1-Ab, H2-Ab, and H3-Ab with homologous and heterologous assay formats; Figure S12: The SDS-PAGE of purified antibodies. Lane M, standard protein markers; Lane 1, the reduction result for H1-Ab; Lane2, the reduction result for H2-Ab; Figure S13: Optimization of ELISA concentration of the coated antigen and antibody. H1-Ab with Hapten3-OVA (a, b) and H2-Ab with Hapten4-OVA (c, d); Table S1: MS/MS conditions; Table S2: Analysis of Sulfonylureas in Real Samples by ELISA and LC-MS/MS.

## Abbreviations

UPLC: ultra-performance liquid chromatography; LC-MS/MS: liquid chromatography-tandem mass spectrometry; DART-MS: real-time mass spectrometry; 2D-MS: on-line two-dimensional liquid chromatography; ELISA: enzyme-linked immunosorbent assay; Ic-ELISA: Indirect competitive enzyme-linked immunosorbent assay; BSA: bovine serum albumin; OVA: ovalbumin; EDC: 1-(3-Dimethylaminopropyl)-3-ethyl carbodiimide hydrochloride; NHS: hydrolyzed protein, N-hydroxylsuccinamide; TMB: freund’s incomplete adjuvant, 3,3′,5,5′-tetramethylbenzidine; DMF: N, N-Dimethylformamide; CB: coating buffer (0.1 mol/L carbonate buffer, pH 9.6); PBS: phosphate buffered solution (0.01 mol/L, pH 7.4); PBST: PBS solution containing 0.5% Tween-20; SDS-PAGE: sodium dodecyl sulfate-polyacrylamide gel electrophoresis; LOD: the detection limit; CR: the cross-reactivity; CV: coefficient of variation; IC50: half of inhibitory concentration.
