# Supplementary material for: Complementary Strategy Enhancing Broad-Specificity for Multiplexed Immunoassay of Adulterant Sulfonylureas in Functional Food
Source: Biosensors (Basel). 2022 Aug 2;12(8):591. doi: 10.3390/bios12080591 (PMC9405802; doi:10.3390/bios12080591)
Supplement: Supplementary file 1 [file biosensors-12-00591-s001.zip › biosensors-1792606-supplementary.pdf]

Supporting information

Complementary strategy enhancing broad-specificity for multiplexed immunoassay of adulterant sulfonylureas in functional food

Zhaodong Li [a†], Haihuan Xie [b†], Yingying Li [c], Xing Shen [a], Xiangmei Li [a], Yi Lei [d], Xiaojun Yao [e], Anastasios Koidis [f], Yingju Liu [a], Hongtao Lei [a*]

[a] Guangdong Provincial Key Laboratory of Food Quality and Safety / National-Local Joint Engineering Research Center for Machining and Safety of Livestock and Poultry Products, South China Agricultural University, Guangzhou 510642, China

[b] Wuzhou Institute for Food and Drug Control, Wuzhou, 543099, Guangxi, China

[c] Wuzhou Product Quality Inspection Institue, Wuzhou, 543001, Guangxi, China

[d] Guangdong Institute of Food Inspection, Zengcha Road, Guangzhou 510435, China

[e] Dr.Neher's Biophysics Laboratory for Innovative Drug Discovery, State Key Laboratory of Quality Research in Chinese Medicine, Macau Institute for Applied Research in Medicine and Health, Macau University of Science and Technology, Macau 999078, China

[f] Institute for Global Food Security, Queen's University Belfast, 19 Chlorine Gardens, Belfast, BT9 5DJ, UK

† These authors contributed equally to this work.

* Corresponding author.

Tel.: +8620 8528 3925; Fax +8620 8528 0270. E-mail: hongtao@scau.edu.cn (H. Lei)

1. Synthesis of Hapten 1

To a solution of 4-(2-aminoethyl)benzenesulfonamide (1 g, 5 mmol) in DMF (20 mL) was added Di-tert-butyl decarbonate (1.3 g, 6 mmol) and triethylamine (0.82 mL) with constant stirring at room temperature. After being stirred for 1 h, water (40 mL) was added to the reaction mass. The solid obtained was filtered, washed with water, and dried to afford tert-butyl-4-sulfamoylphenethylcarbamate (TBS).

In a reaction vessel, TBS (0.9 g, 3 mmol), potassium carbonate (0.62 g, 4.5 mmol), and acetone (20 mL) were mixed and heated to 50-55°C. Cyclohexyl isocyanate (0.56 g, 4.5 mmol) was added slowly to the mixture. After keeping stirring for 3 h at the same temperature, the mixture was cooled to room temperature. Water (60 mL) was added, and the mass was stirred for 30 min. The mixture was filtered. The pH of filtrates was adjusted between 5 to 6 using diluted hydrochloric acid. Solid obtained was filtered and dried to give tert-butyl 4-(N-(cyclohexylcarbamoyl)sulfamoyl) phenethyl carbamate (Hapten 1-BOC) (**Figure S1**).

Hapten 1-BOC (0.85 g, 2 mmol), dichloromethane (3 mL), and trifluoroacetic acid (1 mL) were charged with constant stirring at room temperature. After being stirred for 30 min, Ether (20 mL) was added to the mixture, and the solid obtained was filtered to give 4-(2-Aminoethyl)-N-(cyclohexylcarbamoyl)benzenesulfonamide trifluoroacetate (Hapten 1), ESI-MS: m/z $[M-H]^-$ 323.9 **(Figure S2)**.

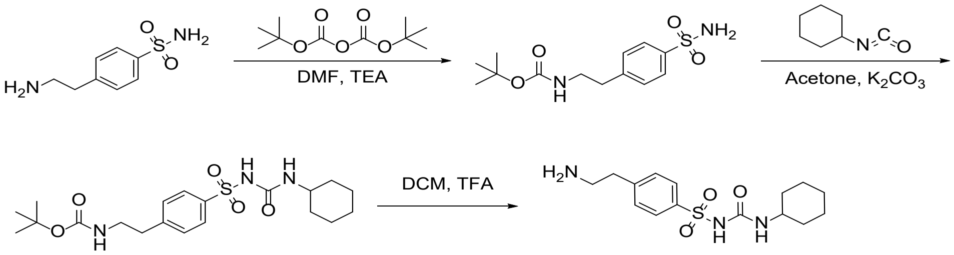

Figure S1. The synthetic routes of Hapten 1

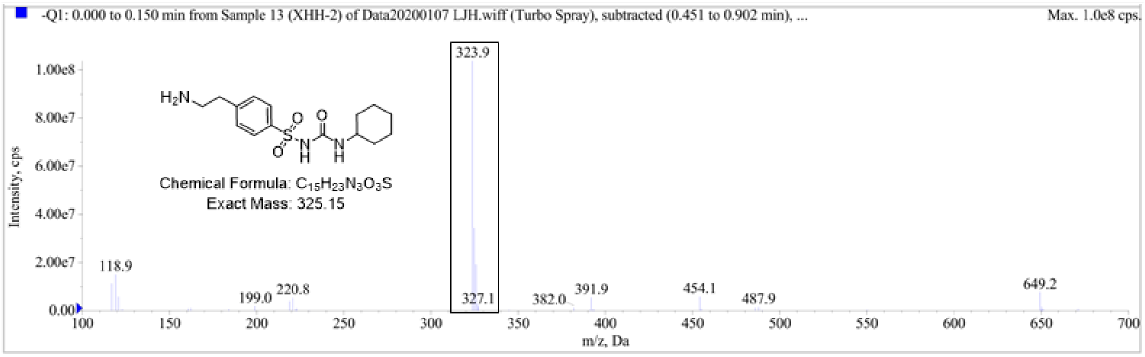

Figure S2. The result of ESI-MS analysis (negative) for Hapten1

2. Synthesis of Hapten 2

Weigh 4-methylbenzenesulfonyl isocyanate (1 g, 5 mmol), glycine methyl ester hydrochloride (1.9 g, 15 mmol) and anhydrous triethylamine (4.15 g, 30 mmol), and dissolve in 10 mL of anhydrous epoxy cyclohexane. The mixture was heated to reflux for 3 h. After the mixture is cooled, spin off the solvent, add 20 mL of 2 mol/L hydrochloric acid solution, mix with the residue, and extract with 40 mL of ethyl acetate. The ethyl acetate was spin-dried and purified by a silica gel column. Dissolve the purified product with 20 mL of methanol, and then add 1 mL of 2 mol/L NaOH solution. The reaction was stirred at 70 ~ 80 °C for 1 h. After the completion of the reaction was monitored by TLC, the pH was adjusted to 5 ~ 6 with hydrochloric acid. The solvent was spin-dried, the methanol was added to dissolve the solid, and the filter removed the insoluble matter. The filtrate was spin-dried again to obtain Hapten 2, ESI-MS: m/z $[M-H]^-$ 270.7. (**Figure S3 and S4**)

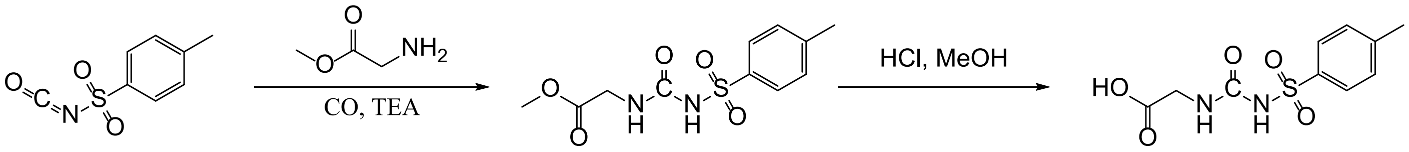

Figure S3. The synthetic routes of Hapten 2

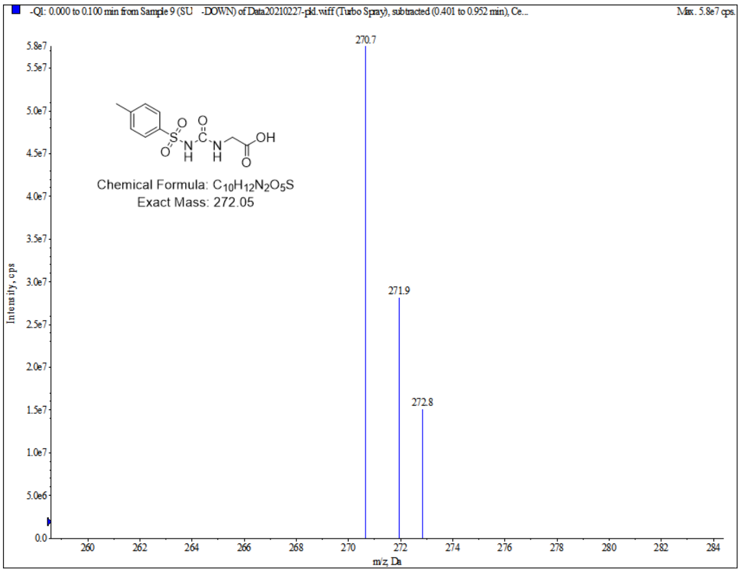

Figure S4. The result of ESI-MS analysis (negative) for Hapten 2

3. Synthesis of Hapten 3

In a reaction vessel, N-(4-sulfamoylphenyl)acetamide (2.14 g, 10 mmol), potassium carbonate (2.07 g, 15 mmol), and acetone (30 ml) were mixed, heated to 50-55°C and reflux for 6 h. when the mixture cool to room temperature, add cyclohexyl isocyanate (1.89 g, 15 mmol) to the mixture, stir and react at room temperature for 1 h. After 8 hours of reaction with reflux, cooling in an ice bath, suction filtration, the white solid obtained was added to 100ml of distilled water, and the insoluble matter was removed by suction filtration. The filtrate was adjusted to pH 5-6 with concentrated hydrochloric acid, and a white solid was precipitated, which was filtered with suction and dried to obtain N-(4-(N-(cyclohexylcarbamoyl)sulfamoyl)phenyl)acetamide (CSPA). CSPA (1.70 g, 5 mmol), NaOH (0.60 g, 15 mmol), and water (5 ml) were charged and mixed in a reaction vessel. After reflux for 10 hours, adjust the pH of the reaction solution to 5-6. A white solid precipitated out, filtered, and dried to obtain Hapten 3, ESI-MS: m/z $[M-H]^-$ 296.0. (**Figure S5 and S6**)

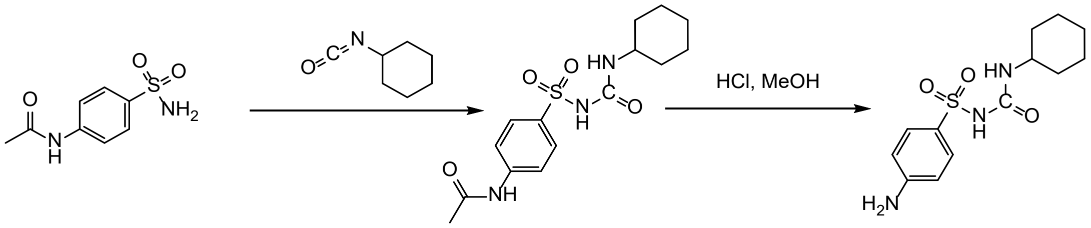

Figure S5. The synthetic routes of Hapten 3

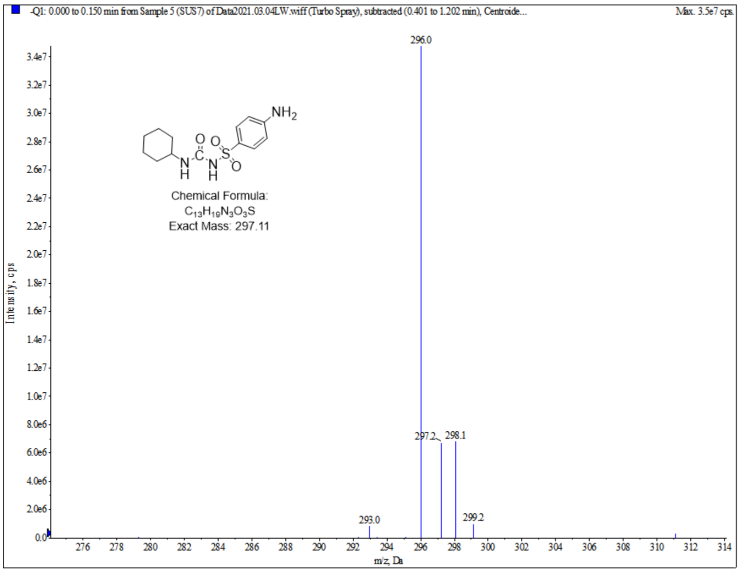

Figure S6. The result of ESI-MS analysis (negative) for Hapten 3

4. Synthesis of Hapten 4

To solution ethyl tosylcarbamate (0.243 g, 1mmol) in 5 mL of toluene and heat to reflux. The tert-butyl (4-aminobutyl)carbamate (0.226 g, 1.2 mol)was mixed with 5 mL of toluene, the mixture was added dropwise to the reaction, and the reaction was refluxed for 6 h. After the reaction, cooled to room temperature, the toluene was spin-dried, 10 mL of ethyl acetate was added, and the product was purified by silica gel column chromatography to obtain Hapten 4 with a tert-butyl ester protecting group (Hapten4-BOC).

Dissolve the Hapten 4-BOC with 10 mL methanol, then add 3 mL concentrated hydrochloric acid, stir and react overnight at room temperature to remove BOC. The solution was spin-dried and obtained the Hapten 4, ESI-MS: m/z $[M-H]^-$ 283.8.

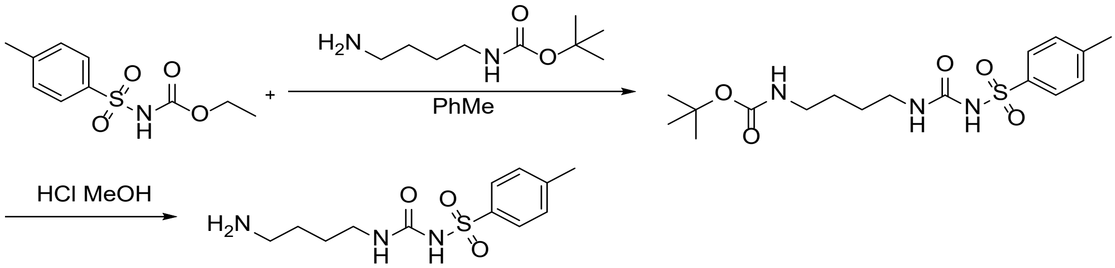

Figure S7. The synthetic routes of Hapten 4

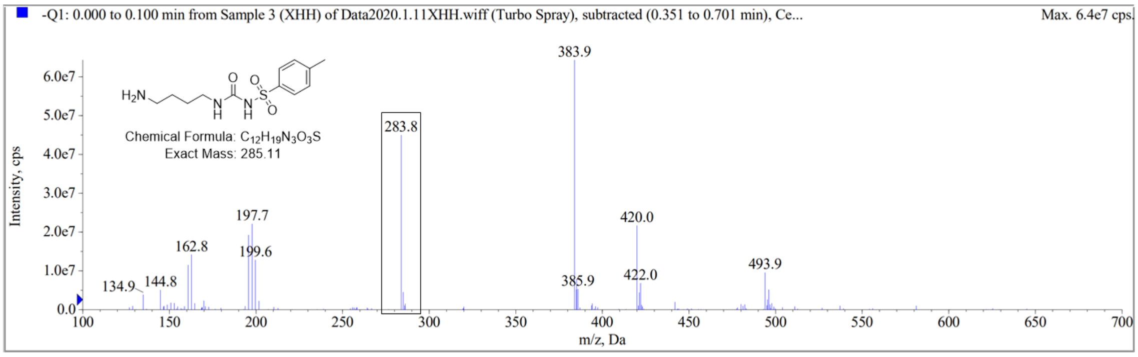

Figure S8. The result of ESI-MS analysis (negative) for Hapten 4

5. General ELISA procedure

The ELISA polystyrene plates were coated with coating antigens (100 μL/well, dissolved in CB) and incubated at 37 °C overnight. The coated plates were washed twice with PBST and blocked with blocking solution (120 μL/well) at 37 °C for 3 h. When finished blocking, the plates were dried at 37 °C for 1 h and stored at 4 °C until use. Sulfonylureas standards or samples solution (50 μL/well) was added, followed by adding H1-Ab or H2-Ab dilutions (50 μL/well) and then incubated at 37 °C for 40 min. Once the antigen competition or antibody binding process is over, washing plates five times and goat anti-rabbit (IgG-HRP, 0.5 mg/mL) diluted by 4000-times with PBST was added to wells (100 μL/well). After another 30 min of incubation at 37 °C and five times washing, TMB solution (100 μL/well) was added and incubated for 10 min. Finally, stop reagents (50 μL/well) were added to the reaction. The microplate reader was used to record the absorbance at the wavelength of 450 nm.

華南農業大學
South China Agricultural University

Results of ethical review of animal experiments

NO：2020e009

Experiment Item	Research on the Key Technology of Multidimensional Inspection of the Authenticity of Important Food			
Application number	2020E009			
Comments on conservation of experimental animals	·All the experimental rabbits used in this experiment came from an experimental animal center legal license. The type, quantity, and grouping of rabbits were conformed to the 3R principle.			
Comments on welfare evaluation of laboratory animals	This experiment was carried out in a laboratory with a license for experiment animals, which was confirmed to the welfare principle.			
Comments on ethical and moral	The animals were euthanized after the experiment.			
Comments on comprehensive scientific evaluation	This experimental study has scientific significance.			
Time of experiment animal type and quantity	Date: 2020-09-01 to 2020-12-01.<br>Experimental animal: regular New Zealand white rabbits.<br>Quantity: 10 males and 10 females			
Comments of the ethical reviewer	Agree			
	Reviewer	Wei Huang	Review Date	2020-08-16
Comments of the ethical reviewer	Agree			
	Reviewer	Rangcai Yu	Review Date	2020-08-16
Final comments of the director (or deputy director)	Agree			
	Reviewer	Zhonghua Liu	Review Date	2020-08-16

Experimental Animal Ethics Committee of
South China Agricultural University
Date: 2020年8月26日

Figure S9. Ethical review of animal experiments

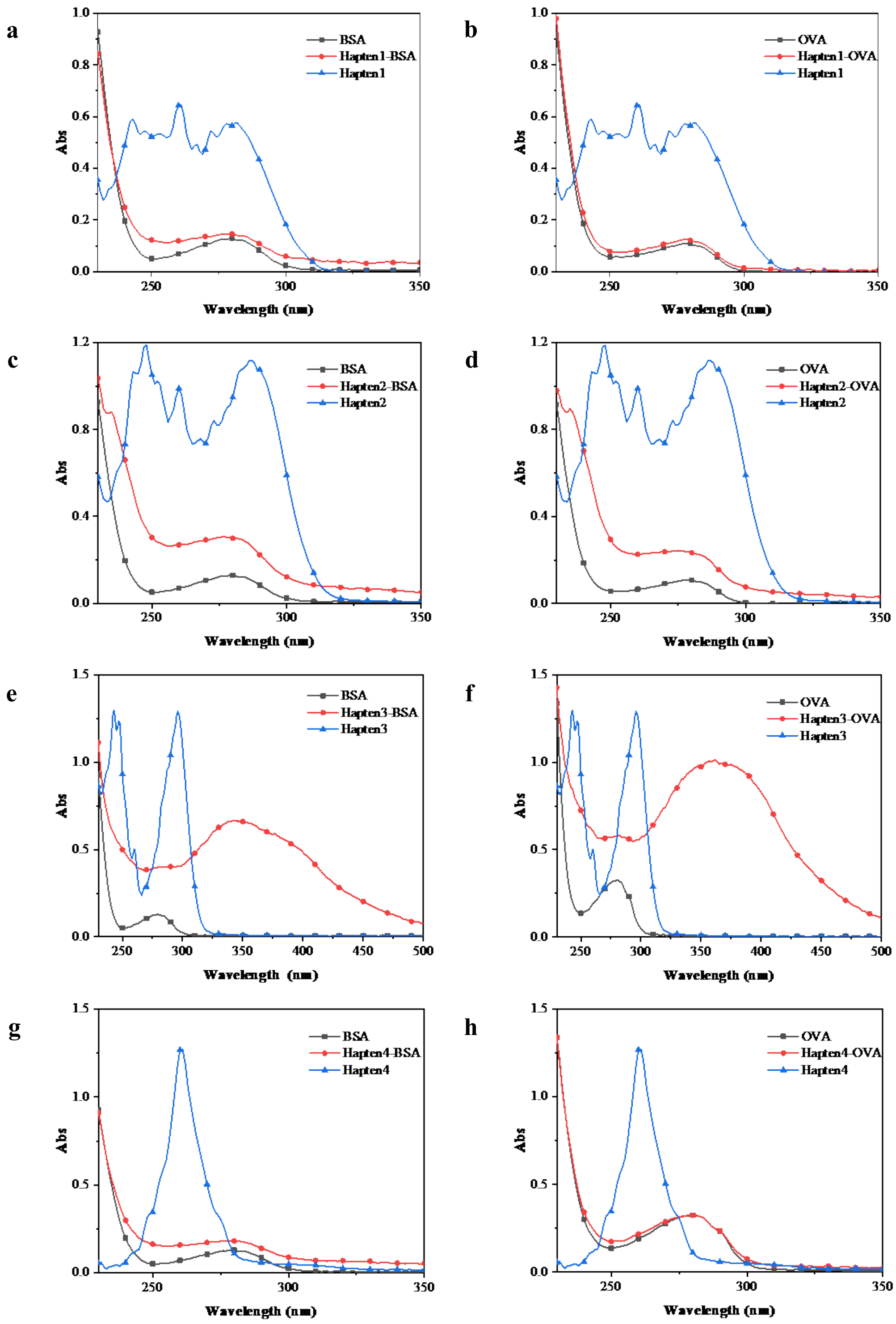

Figure S10. The UV-VIS spectroscopy of Hapten1-BSA (a), Hapten1-OVA (b), Hapten2-BSA (c), Hapten2-OVA (d), Hapten3-BSA (e), Hapten3-OVA (f), Hapten4-BSA (g), Hapten4-OVA (h), and corresponding carrier protein and hapten.

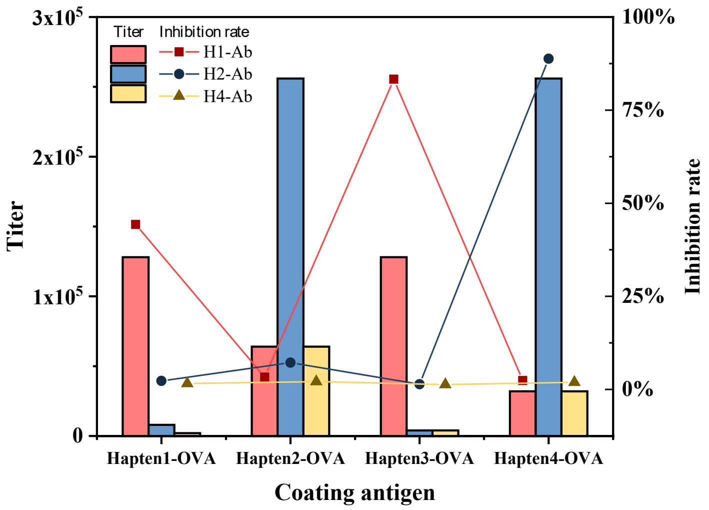

Figure S11. Titer and inhibition test for H1-Ab, H2-Ab, and H3-Ab with homologous and heterologous assay formats.

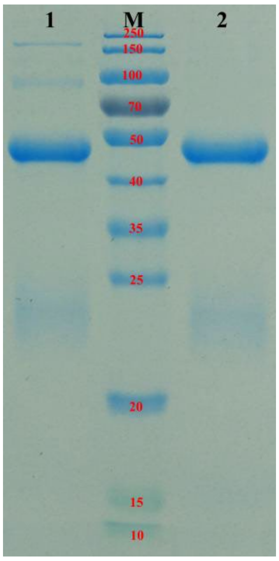

Figure S12. The SDS-PAGE of purified antibodies. Lane M, standard protein markers; Lane 1, the reduction result for H1-Ab; Lane2, the reduction result for H2-Ab.

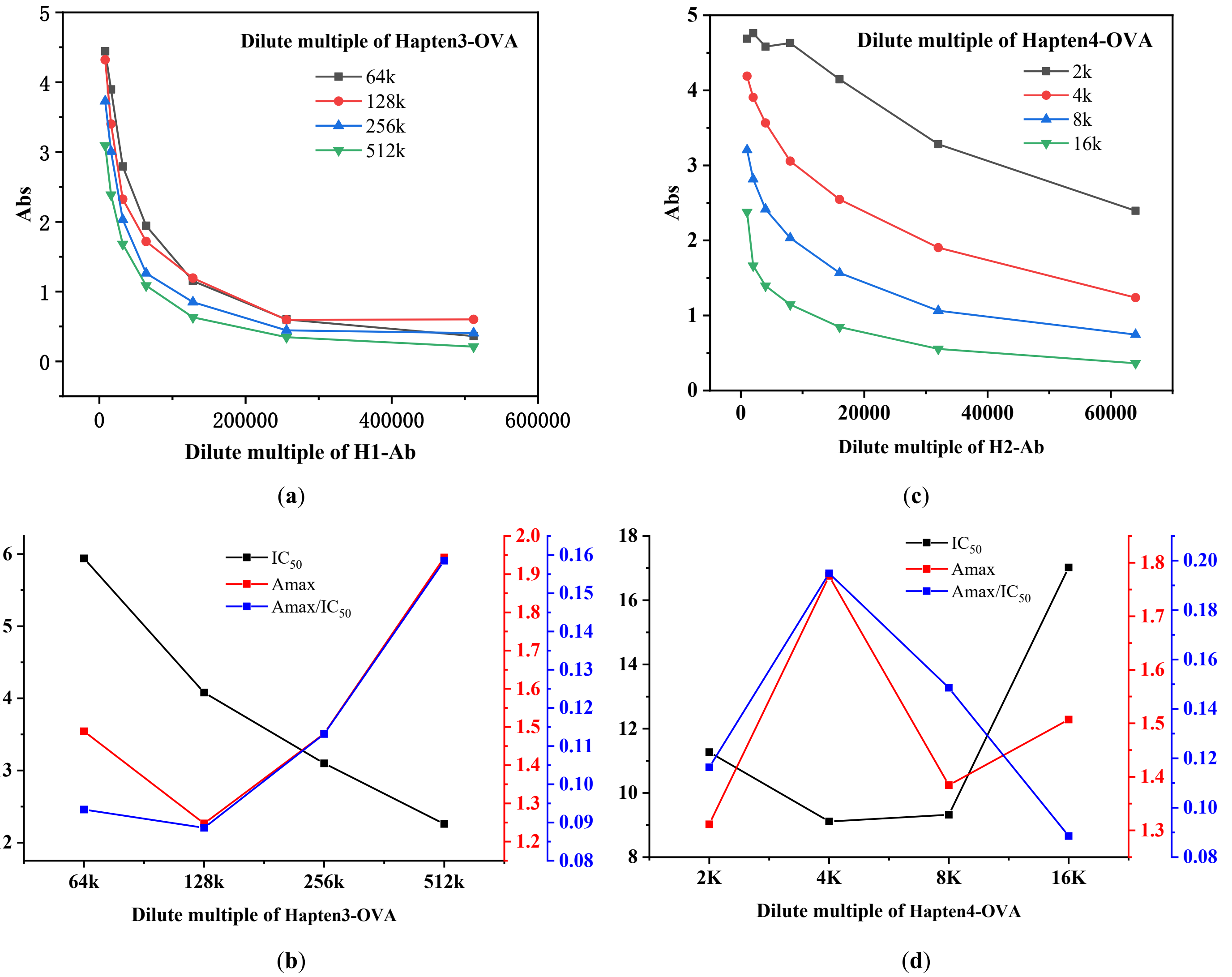

Figure S13. Optimization of ELISA concentration of the coated antigen and antibody. H1-Ab with Hapten3-OVA (a, b) and H2-Ab with Hapten4-OVA (c, d).

Table S1. MS/MS conditions.

Analyte	Precursor ion /(m/z)	Product ion /(m/z)	DP / eV	CE / V
Glyburide	494.1	368.9*	90.00	17.00
	494.1	168.8	90.00	38.42
Glipizide	446.2	320.9*	80.00	18.00
	446.2	437.0	80.00	19.13
Glimepiride	491.2	352.1*	110.00	17.00
	491.2	125.9	110.00	34.28
Gliquidone	528.1	402.9*	93.00	19.00
	528.1	386.1	93.00	28.25
Tolbutamide	270.9	171.9*	66.00	16.00
	270.9	155.0	66.00	25.89
Gliclazide	324.1	110.0*	81.00	24.00
	324.1	127.1	81.00	23.00
Acetohexamide	325.1	243.0*	94.00	15.00
	325.1	119.0	94.00	34.00
Carbutamide	272.3	156.0*	59.00	19.80
	272.3	74.1	59.00	15.00
Tolazamide	311.9	115*	86.00	22.00
	311.9	91.1	86.00	40.00
Chlorpropamide	277.0	111.0*	56.40	42.00
	277.0	174.8	50.74	20.00
Glibornuride	367.0	170.2*	63.08	20.00
	367.0	152.1	75.98	26.00

*Quantitative ion

Table S2. Analysis of Sulfonylureas in Real Samples by ELISA and LC-MS/MS.

		S1	S2	S3	S4	S5	S6	S7	S8
**ELISA1**	**Observed concentration**	ND	ND	ND	ND	ND	ND	ND	ND
**ELISA2**	**Observed concentration**	ND	ND	ND	ND	ND	ND	ND	ND
**LC-MS/MS**	**Glimepiride**	ND	ND	ND	ND	ND	ND	ND	ND
	**Glipizide**	ND	ND	ND	ND	ND	ND	ND	ND
	**Glyburide**	ND	ND	ND	ND	ND	ND	ND	ND
	**Gliquidone**	ND	ND	ND	ND	ND	ND	ND	ND
	**Acetohexamide**	ND	ND	ND	ND	ND	ND	ND	ND
	**Tolazamide**	ND	ND	ND	ND	ND	ND	ND	ND
	**Tolbutamide**	ND	ND	ND	ND	ND	ND	ND	ND
	**Carbutamide**	ND	ND	ND	ND	ND	ND	ND	ND
	**Gliclazide**	ND	ND	ND	ND	ND	ND	ND	ND
	**Chlorpropamide**	ND	ND	ND	ND	ND	ND	ND	ND
	**Glibornuride**	ND	ND	ND	ND	ND	ND	ND	ND
